# Real-Time Seismic Data from the Bottom Sea

**DOI:** 10.3390/s18041132

**Published:** 2018-04-08

**Authors:** Xavier Roset, Enric Trullols, Carola Artero-Delgado, Joana Prat, Joaquin Del Río, Immaculada Massana, Montserrat Carbonell, Jaime Barco de la Torre, Daniel Mihai Toma

**Affiliations:** 1 SARTI Research Group, Universitat Politècnica de Catalunya (UPC), 08800 Vilanova i la Geltrú, Spain; xavier.roset@upc.edu (X.R.); enric.trullols@upc.edu (E.T.); carola.artero@upc.edu (C.A.-D.); joana.darc.prat@upc.edu (J.P.); joaquin.del.rio@upc.edu (J.D.R.); immaculada.massana@upc.edu (I.M.); montse.carbonell@upc.edu (M.C.); 2 Instituto Geográfico Nacional C/General Ibáñez de Íbero 3, 28003 Madrid, Spain; jbarco@fomento.es

**Keywords:** earthquake, marine seismometers, data transmission and management systems, inductive communications

## Abstract

An anchored marine seismometer, acquiring real-time seismic data, has been built and tested. The system consists of an underwater seismometer, a surface buoy, and a mooring line that connects them. Inductive communication through the mooring line provides an inexpensive, reliable, and flexible solution. Prior to the deployment the dynamics of the system have been simulated numerically in order to find optimal materials, cables, buoys, and connections under critical marine conditions. The seismometer used is a high sensitivity triaxial broadband geophone able to measure low vibrational signals produced by the underwater seismic events. The power to operate the surface buoy is provided by solar panels. Additional batteries are needed for the underwater unit. In this paper we also present the first results and an earthquake detection of a prototype system that demonstrates the feasibility of this concept. The seismometer transmits continuous data at a rate of 1000 bps to a controller equipped with a radio link in the surface buoy. A GPS receiver on the surface buoy has been configured to perform accurate timestamps on the seismic data, which makes it possible to integrate the seismic data from these marine seismometers into the existing seismic network.

## 1. Introduction

Variations in real-time seismicity provide knowledge of the state of local and regional stresses in the short and medium term, essential information to study the potential seismic risk that may affect infrastructures and population located in the area. Recent seismic activity in 2013 (possible induced by a gas tank) on the coast of Vinaròs, or the intense underwater seismic activity associated with the eruption of El Hierro in the archipelago of the Canary Islands (2011–2012), shows the importance of controlling seismic events located in the sea that are not covered by the terrestrial monitoring networks.

Monitoring the regional seismic events in real time makes it possible to achieve fast estimation of actual earthquake scales, since the measured seismicity directly gives us the true size of the earthquake. In the last decade, various technologies have been proposed and tested to address this challenge, such as cabled Ocean Bottom Seismometers (OBSs) [1,2,3,4], OBSs acoustically linked to surface buoys or surface vehicles [5,6] and free-floating buoys [7]. Although a permanent array of wired OBSs can be considered as the best technology for seismic surveys, having practically unlimited power and data transmission bandwidth, it is very expensive to implement and its deployment is restricted to the locations where such cable observatories can be deployed. More affordable solutions are the OBSs acoustically linked to surface buoys or surface vehicles. However, the standalone ocean bottom broadband stations, implementing an acoustic link between the OBS and the surface, are limited by the acoustic communication latency, power consumption, and bandwidth.

Within this context, we developed and implemented a new technology for standalone ocean bottom broadband systems. First, an overview of the challenges for underwater monitoring of the regional seismic events in real time is presented. Section 2 focuses on the design philosophy of the standalone ocean bottom broadband system including the seafloor unit, the surface buoy, and the mooring and the inductive communication system; it includes the dynamic simulations of the prototype for deployment near the OBSEA observatory at a depth of 20 m and in the Alboran Sea (Western Mediterranean) at a depth of 250–300 m. The actual deployment of the standalone ocean bottom broadband station near the OBSEA observatory and the results are discussed in Section 3. The results include the registration of a seismic event located in the Hautes-Pyrénées, of magnitude 3.7, that occurred on 28 October at 19.06 UTC. Finally, the conclusions drawn are presented in Section 4.

## 2. Standalone Broadband Ocean Bottom System

In this paper we describe a moored OBS system, with continuous telemetry through the mooring line and buoy to the shore station, capable of providing good azimuth observations of regional seismic events. The real-time observations are useful to determine the magnitude and the hypocentre of the regional seismic events. Therefore, the moored OBS system should detect seismic events of low seismicity (1.5 < M < 5), located at depths up to 100 km, and the nominal natural period of 1 second. This requires a bandwidth of 0.5 to 30 Hz with 18 bits of resolution, with a signal to noise ratio above 60 dB, and a communication data loss rate less than 10%. Hence, the degraded seismic data transfer is assumed, as short-term packet loss may occur in moored OBS system [8].

**Figure 1:**
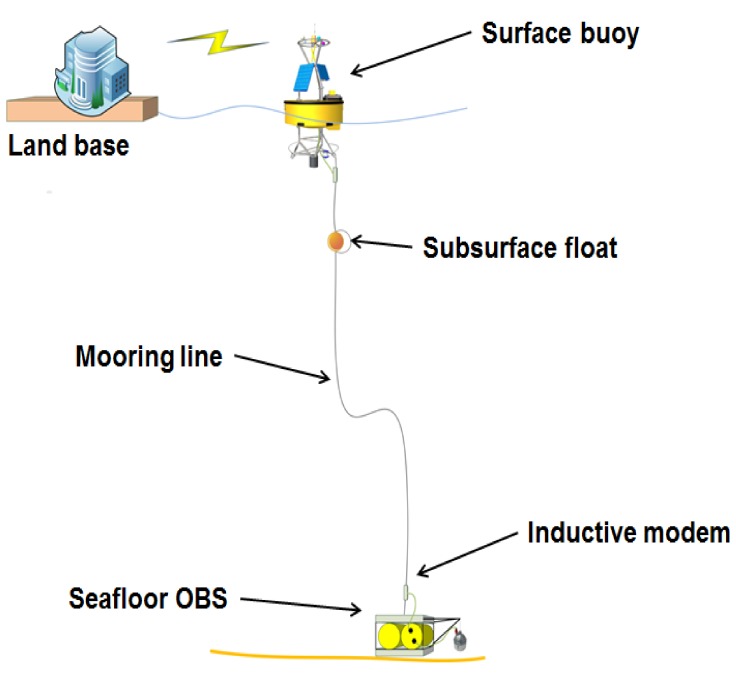
Overview of the Real-Time Seismic Coastal Ocean Station.

An anchored OBS has been built and tested to acquire regional and distant seismicity in near real time. The overall latency of the system is low (a few tens of seconds), and is dominated by the time to gather a group of data to telemeter, the time to send the data through the inductive link, and the time to send the data through the GPRS or satellite network. The first deployment was done in June of 2017, at a depth of 20 m, 2 nautical miles off coast of Vilanova i la Geltrú (Barcelona), near the OBSEA underwater observatory [9]. The design consists of a seafloor OBS with a broadband sensor unit and a surface buoy connected to a cable with an intermediate buoy (Figure 1). The validation of this prototype seismic system was done, comparing the data with the existing OBSEA seismometer [4]. The seismic system continuously transmits data to a controller in the surface buoy, at a rate of 1000 bps through an inductive link provided by two inductive modems [10] attached to the mooring line as illustrated in Figure 1. Next, the controller in the buoy forwards the data to the land station through a GPRS link, if the system is placed offshore (tens of kilometres), or through satellite communications, if the system is placed in a far offshore ocean environment. Seismic data from the moored OBSs can be integrated into the existing seismic data network thanks to the GPS receiver on the surface buoy, which is configured to timestamp all the data coming from the seafloor seismometer. The seismic data is continuously recorded in the vertical channel with a triggering algorithm to switch to 3 components for special events. On demand, the users can also request the data packet of all three channels for short time periods.

### 2.1. The Seafloor Unit

**Figure 2:**
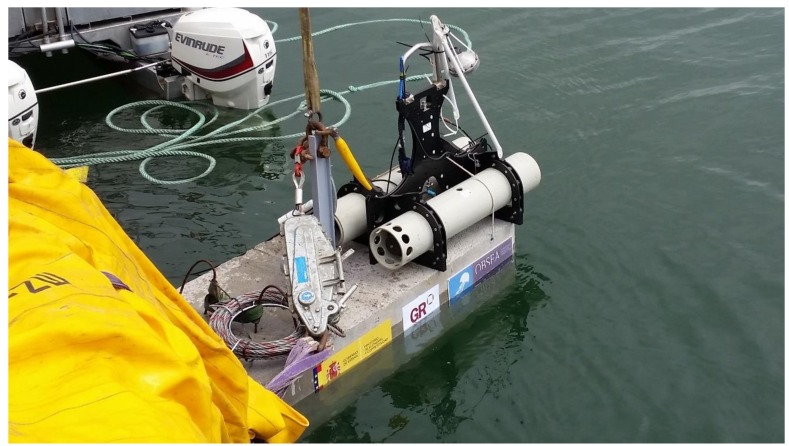
The prototype ocean bottom broadband unit.

**Figure 3:**
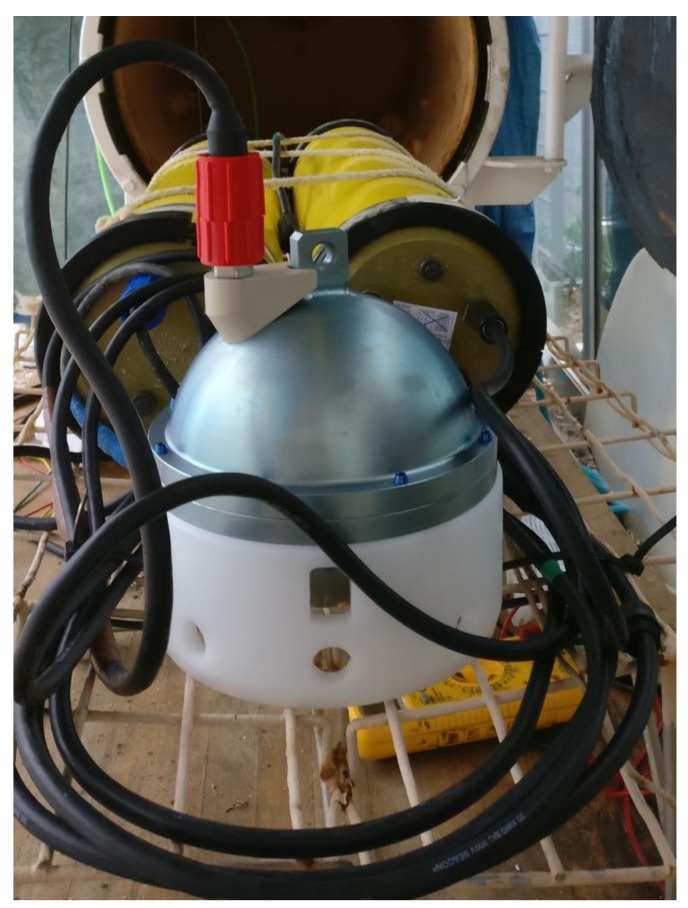
The Ocean Bottom Seismometer (OBS) with Güralp 6TC seismometer at pressure test. The bottom case of the seismometer is grooved to evacuate exceeding material for a successful coupling with the seafloor.

The seafloor unit consists of an anchor, a seismometer containing 3 geophones (one in each orthogonal axis), and the associate electronics and the batteries (Figure 2). When the equipment reaches the seabed, the arm that supports the seismometer is tilted and separates the seismometer from the anchor and next the seismic sensor is positioned on the seabed, next to the anchor with the acquisition and power unit, by only the signal and power cable. The broadband seismometer is a Güralp 6TC OBS Gimbal with a sensitivity of 1630 V/m/s for each geophone and with a cut-off of at 0.033 Hz for detecting low frequency Earth movements. As illustrated in Figure 3, the bottom case is grooved to evacuate exceeding material like sand, mud, sludge, or other kind of sediments, getting a successful coupling with the seafloor. A centring of the Güralp 6TC OBS masses is required when the seismometer is first deployed on the seafloor. This operation can be done from the land base at any time through the bidirectional communication link provided by the inductive and the RF link. 

**Figure 4:**
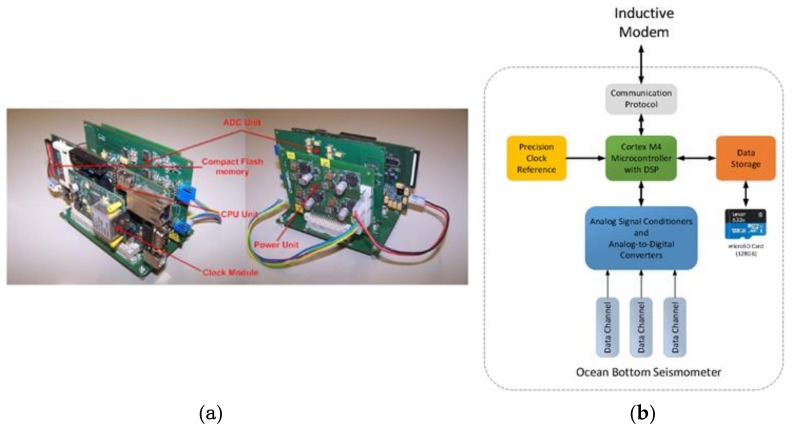
(**a**) Electronic acquisition system and (**b**) block diagram of OBS electronics. The Inductive modem interface connects to the OBS via a serial line.

The seismic data of the Güralp 6TC OBS is acquired and managed by an acquisition system (Figure 4), which consists of five main blocks: analogue-to-digital conversion module, micro-controller and data storage module, power regulation module, time base module, and inductive communication module as illustrated in Figure 4.

The prototype broadband OBS is a modification of the autonomous one [8] with the addition of an inductive modem and its associated interface processor. A lithium-ion battery pack is built and used as the main power supply. The micro-controller and storage module is based on a LPC 4357 microcontroller and a 64 GB microSD memory card for data storage (approximately 1 year of raw seismic data). This module is in charge of configuration of the ADC module selecting the sampling rate and low power operations. The acquisition software designed acquires data continuously from input channels through a QSPI (Queued Serial Peripheral Interface) bus, performs local timestamping using the integrated RTC (Real-Time Clock), and stores the data in the microSD memory card. In addition to the seismic measurements, the internal temperature and humidity measurements are also performed. The data acquisition and storage system has been optimized to acquire seismic events in the domain of seismology. The data logger has a dynamic range of all the channels of 129 dB, slightly below the ADC dynamic range at the same sampling rate (130 dB@103 Hz). The resolution of the system is about 21.4 bits due to noise and the random noise is about 1.1 LSBs which corresponds to about 320 nV. A channel crosstalk of −147 dB shows a good PCB design and EMI immunity. 

The acquired measurements are timestamped and organized into fixed length miniSEED files and stored on a microSD card. In normal mode, sub-samples seismic data acquired from the vertical axis of the seismometer is transmitted every ten seconds to the shore station through the inductive communication using a Sea-Bird UIMM module and the surface link. If required by the operators, the system can transmit historic packages of seismic data from all 3 axes at full resolution. Furthermore, this module carries out the following functions: time synchronization with a GPS signal, clock drift calculation, and low power operation modes. A lithium-ion battery pack is used as the main power supply and is enough for 6 months in service collecting data.

### 2.2. Surface Buoy

**Figure 5:**
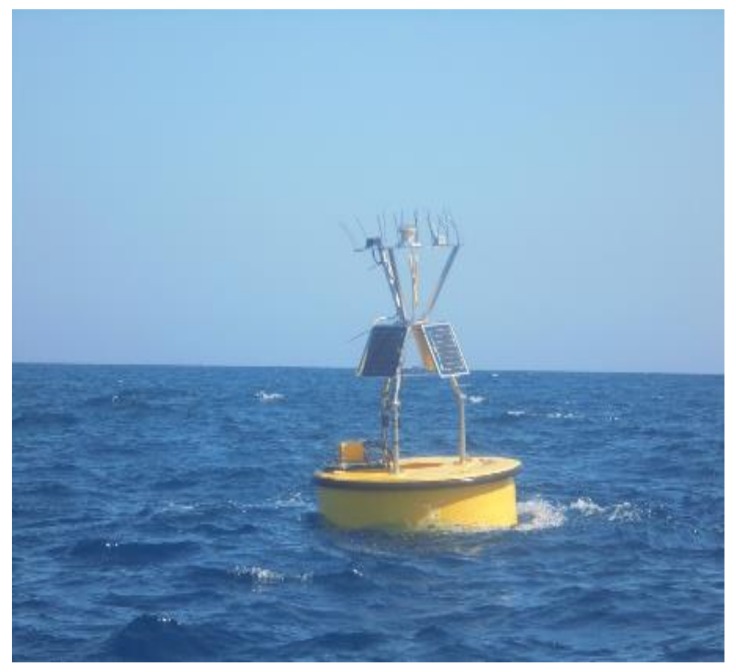
The surface buoy.

In the design of the surface buoy, multiple factors have been taken into account. Therefore, the buoy has been built to support the weight and tensions of the connection cable that links it to the seafloor unit in different sea and weather conditions. Moreover, the buoy is designed to be large enough to contain electronics for collecting data, communication devices, and energy supply. Finally, the surface buoy built for the moored OBS is portable and easily deployed by scientists unfamiliar with the deployment of marine seismometers and also using boats not fully equipped for research. In the above scenario, the chosen buoy is a 2 m diameter toroidal buoy EMS 2.0 (Figure 5) [11].

This buoy includes four solar panels (4 × 12 V/32Wp @ 92Wh/d) of 440 × 460 × 2 mm, two solar charge controllers and sealed lead-gel batteries 12 V/120 Ah—which supply and store the required energy—an AirLink Raven RV50 modem, and a system controller based on Raspberry Pi with a Sea-Bird UIMM for inductive communication with the seafloor OBS.

### 2.3. The Mooring and Inductive Communication System

Prior to the deployment two different models were proposed and the dynamics of the systems were studied numerically in order to design optimal materials, cables, buoys, and connections in these critical marine climate conditions. The final deployment of this system is envisioned for the Alboran Sea (Western Mediterranean), which is an active seismic area at a depth of 250–300 m, where simulations have been done for this location.

A study of meteorological conditions at the Carboneras fault has been done to get the main values for the wind, waves, and current in two cases: normal (or more usual) meteorological conditions as well as in extreme conditions. The OrcaFlex software under academic license N1594 has been used to simulate the dynamics of two different models of the seismometers mooring under these realistic weather conditions.

#### 2.3.1. Mooring Configurations

**Figure 6:**
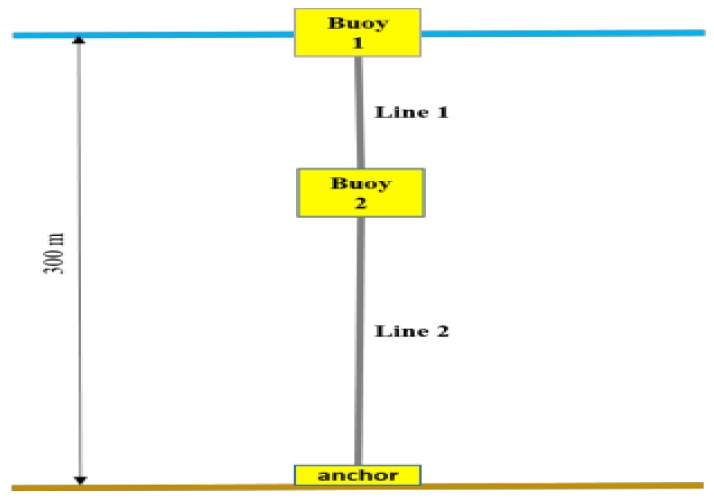
Schematic vertical view of the models.

The proposed design consists of a seabed unit, an intermediate underwater buoy, and a surface buoy (see Figure 6). The intermediate buoy supports part of the weight of the cable minimizing displacements and tensions at the seabed anchorage. The three units are linked with a mooring/communication cable and two options, using a steel cable or a combination of steel cable plus elastomer, have been studied. Mooring cables have to support communication and data transfer. In Model 1 the mooring cable is a plastic-jacketed steel wire rope. Their two ends are connected to seawater ground, closing the electrical circuit. The electrical circuit thus created allows direct injection of data or its transfer using a magnetic field. In Model 2 an additional electrical cable around the elastomer is needed. Keeping in mind that the purpose is to maintain the seabed anchorage as stably as possible, a long steel cable or the use of the elastomer in Line 1 are good options. The inverse catenary or elastomer in Line 1 absorbs part of the movements and tensions induced by the meteorological conditions (waves, winds, and currents). In particular, we evaluated three cases of each model where the length of Line 1 is varied (see Figure 6). The ratio of Line 1 is defined as the length of Line 1 in front the depth of the intermediate buoy in the static case (100 m or 20 m, depending on the Model).

As stated above, in order to support the cable weight (Line 2 and partially Line 1), a cylindrical intermediate buoy of 40 kg mass with a buoyancy of 2000 N has been chosen.

A 6 mm section steel cable line (6 × 19) has been chosen. This is the minimum size that supports the loads in extreme conditions. The selected elastomer is made of natural rubber with cord diameter 35 mm, Shore A, with 300% elongation and hardness of 45 or 60 [12]. In the open sea, the environmental conditions of currents and waves are the main environmental loads acting on the surface buoy, but the load due to the wind cannot be ignored, mainly because of the resistance offered by the solar panels. The wind rose of the “Cabo de Gata” and the maximum wave have been considered to design the system.

#### 2.3.2. Numerical Simulations

The simulations were performed with OrcaFlex 9.3c [13], which is a marine dynamics software implemented by Orcina for the static and dynamic analysis of several offshore systems. OrcaFlex facilitates a fast and accurate analysis of different kind of cables under wave loads and currents and externally forced movements. It is a 3D software of finite elements in a non-linear time domain capable of arbitrarily handling large deflections from the initial condition. OrcaFlex models use orthogonal coordinates with the origin at the sea surface (in the absence of current, wind, and waves). Axis *x* and *y* are horizontal and *z* vertical (negative downwards). Deep sea water (300 m) is considered for the two models (see Table 1). The analyses have been performed for normal and extreme sea conditions (see Table 2). The normal case considers normal sea conditions while the extreme case considers very bad and unusual weather conditions. In order to consider the worst scenario, waves, wind, and current are imposed to have the same direction and sense (*x* positive axis) [14].

Dynamic simulations have been done using an implicit integration method with a fixed step size. The segment length of Line 2 is 1 m whereas in Line 1 it is 0.1 m and 0.2 m. The first segment on the top needs to be smaller when a big ratio of elastomer is considered with extreme conditions. To study temporal behaviour, simulations have been done lasting a long enough time to overpass the transient. With the transient being 600 s, simulations have been run lasting 20,000 s to ensure the overpass. A step size of 0.1 s has been established, sufficient for the proposals of the present work, small enough to generate correct results.

#### 2.3.3. Simulation Results

Table 3 displays values of the horizontal displacement of the surface buoy (surge), the maximum tensions induced by Line 2 to the anchor, and the inclination angle (referred to the vertical at seabed). The most critical value is the tension supported by Line 2 at the seabed (last column in Table 2). This tension, if large enough, could produce movements and displacements in the anchor and also vibrations that could affect the measurements of the nearby seismometer. In normal conditions, we observe that the range of displacement of the surface buoy (measured with the *x* variable) do not depend on the line ratio, being around 12 m in Model 1 and 8 m in Model 2.

**Figure 7:**
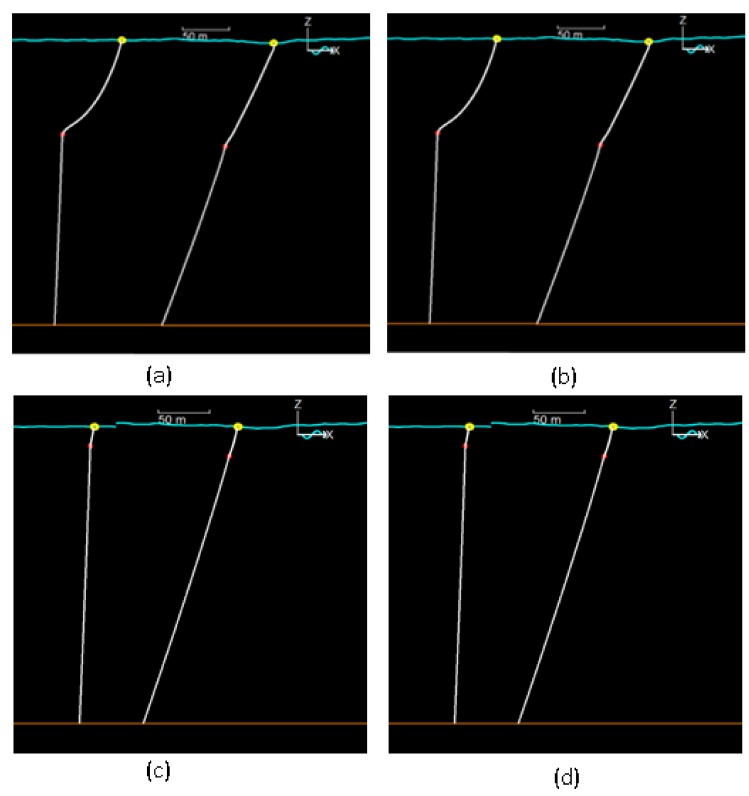
Orcaflex vertical view. Different configurations at a fixed time: Model 1: (**a**) ratio 1.2; (**b**) ratio 1.6. Model 2: (**c**) ratio 0.85; (**d**) ratio 1.2.

The vertical angle of Line 2 remains small and similar in both models, being the maximum 3.5° (see left profile in Figure 7). The tension at the seabed is also small in all cases, with a maximum of 2.2 kN. We can conclude that, in normal conditions, there is no substantial difference between the two models. Taking into account that our purpose is to minimize the angle and the tension in the seabed, we can accept that a bigger ratio gives better results. In extreme conditions, due to the strong tension involved, the Line 1 inverse catenary disappears and acts like a quasi-straight line (see right profile in Figure 7). In Model 1 the surge range of the surface buoy (about 19 m) does not depend on the ratio of lines and only slightly depends on the ratio (36.6–41.4 m) in Model 2. A noticeable difference, regarding maximum surge, Model 1 always shows higher values than Model 2 (about 4 times bigger). The angle of Line 2 is similar in both models under the same meteorological conditions. The proposed design consists of a seabed unit (containing the seismometer), an intermediate underwater buoy (that minimizes displacements and tensions in the system), and a surface buoy (containing the offshore communication system). Under normal conditions there is not a significant difference between models, though in extreme conditions Model 2 gives better results than Model 1, as the seabed tension is about 4 times smaller. Under the studied conditions, the use of the elastomer is a good option in extreme conditions as it reduced the tensions at the anchorage, minimizing the risk of displacement. The buoy has also been over-dimensioned to support extreme conditions. A large anchor and a strong steel cable ensure their stability and the seismometer has been placed as far as possible from the anchor to avoid interferences.

**Figure 8:**
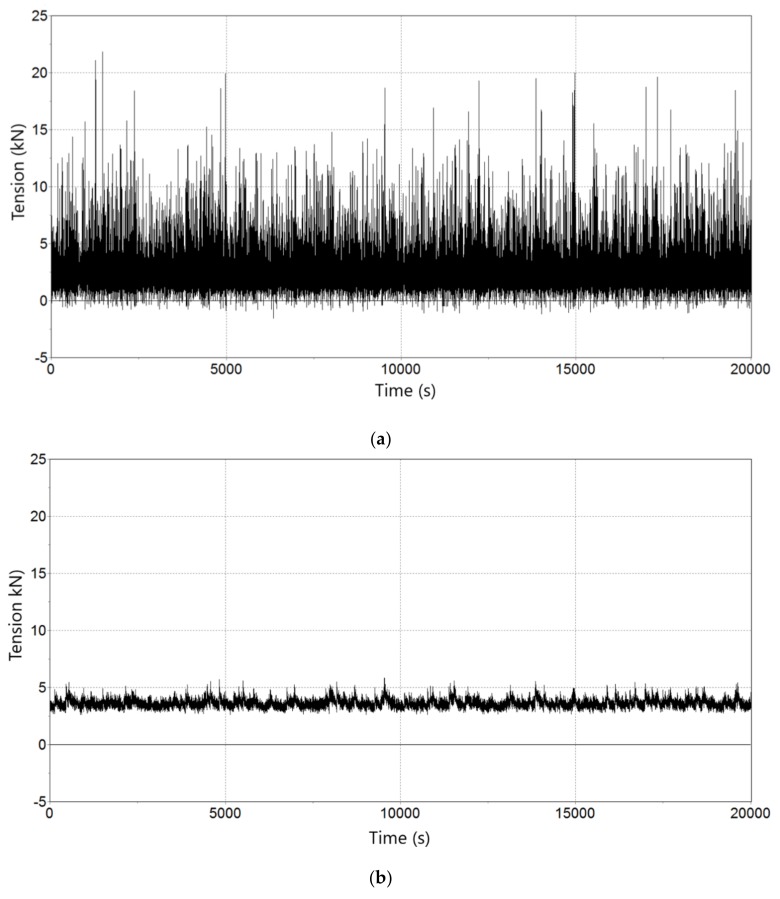
Tension on seabed. Temporal evolution (s) of the tension (kN) of Line 2 on seabed: (**a**) Model 1, ratio 1.4; (**b**) Model 2, ratio 1.2.

**Figure 9:**
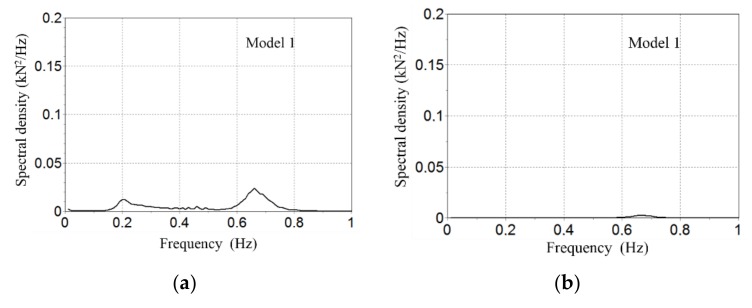

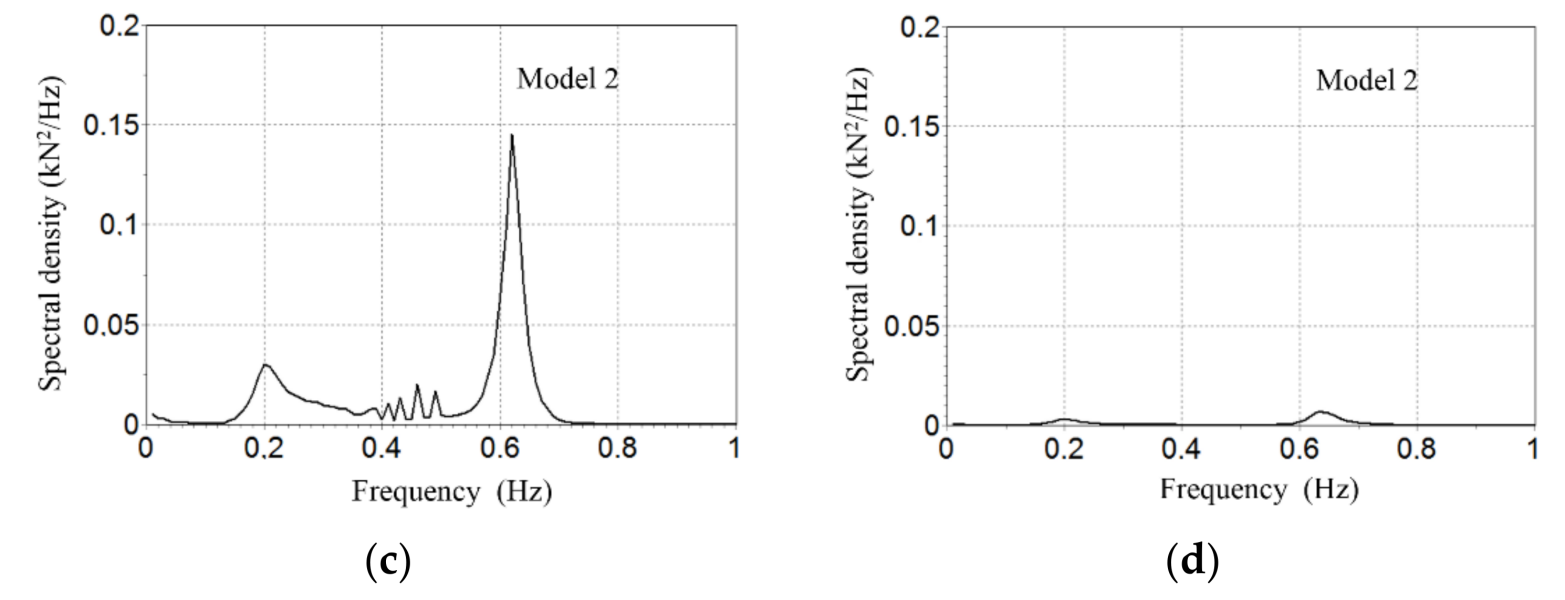
Fourier tension spectral analysis with normal Mateo. Spectral density of the effective tension at the seabed (anchor) vs the frequency (Hs). (**a**) Line 1 ratio of 1.2 (**b**) Line 1 ratio of 1.4; (**c**) Line 1 ratio of 0.85 and (**d**) Line 1 ratio of 1.2.

**Figure 10:**
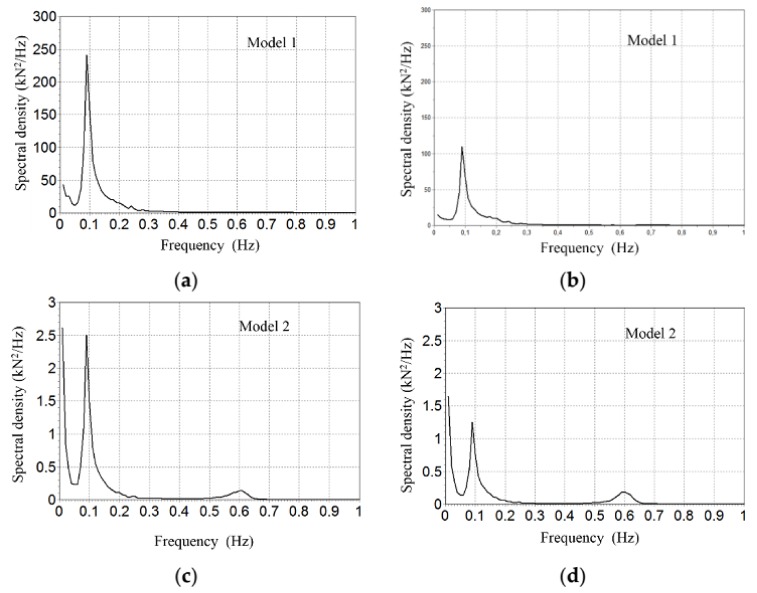
Fourier tension spectral analysis with extreme Mateo. Spectral density of the effective tension at the seabed (anchor) vs the frequency (Hs). (**a**) ratio 1.2 (**b**); ratio 1.4; (**c**) ratio 0.85 and (**d**) ratio 1.2.

There is also a significance difference between maximum tensions at the seabed depending on the model, ranging from 25.9 kN in Model 1 to 7.5 kN in Model 2 (see Figure 8). Figure 9 and Figure 10 show the Fourier spectral density of tensions induced to the seabed using Model 1 and Model 2 respectively (notice the different scales in figures and between them). Dominant frequencies are always smaller than 1 Hz. The spectral analysis is basic in the study of dynamic behaviour. Spectral wave data mainly configures the spectrum, but wind, current, and the mechanical configuration modifies it accordingly, as can be seen in Figure 9. In contrast, with extreme conditions, the spectral density chart shows that main wave frequency is the dominant one (0.09 Hz) (see Figure 10), because the waves are the more impacting environmental condition.

## 3. Results

**Figure 11:**
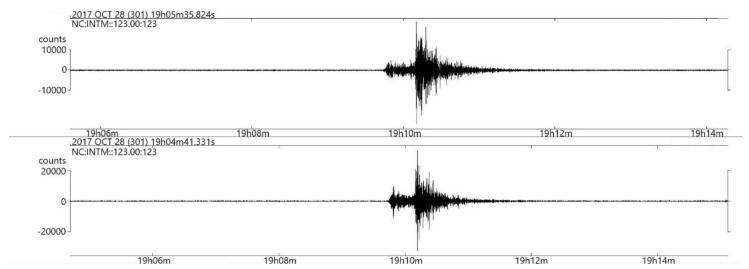
Recording of a seismic event located at Hautes-Pyrénées, of magnitude 3.7, that occurred on 28 October at 19.06 UTC. A band filter between 4 to 10 Hz has been used to filter the raw seismic data.

**Figure 12:**
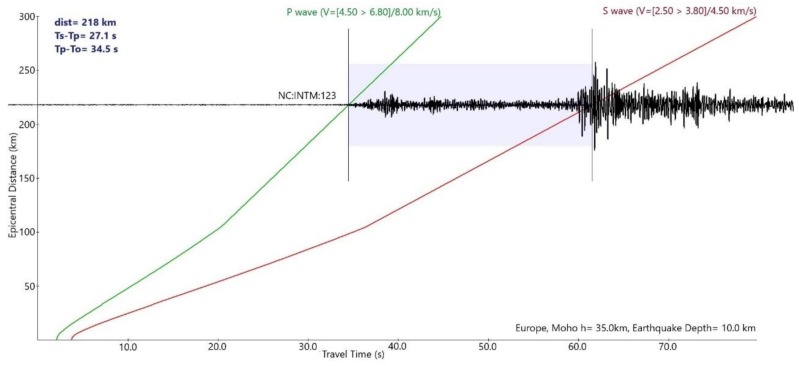
Epicentral distance of the seismic event located at Hautes-Pyrénées.

The results of the validation deployment, done near the OBSEA underwater observatory, have shown that the system is capable of transmitting near real-time seismic data. The telemetry system, via underwater inductive link and surface radio link, is capable of providing a permanent two-way communication link with an acceptable packet loss of less than 3%. Moreover, thanks to the high sensitivity of the broadband marine seismometer, the data collected in these tests has shown that distant earthquakes could be measured with this system. Figure 11 illustrates the seismic data registered by the seismometer from an earthquake located in the Hautes-Pyrénées, of magnitude 3.7, that occurred on 28 October at 19.06 UTC. As depicted in Figure 12, the travel time difference corresponds to an epicentral distance of the earthquake of about 218 km, which is approximately the distance between the place where the OBS has been deployed and where the earthquake occurred in the Hautes-Pyrénées, that has been recorded by all the seismic stations of the Institut Cartogràfic i Geològic de Catalunya (www.icgc.cat) and the cabled OBS installed at OBSEA observatory (www.obsea.es). 

**Figure 13:**
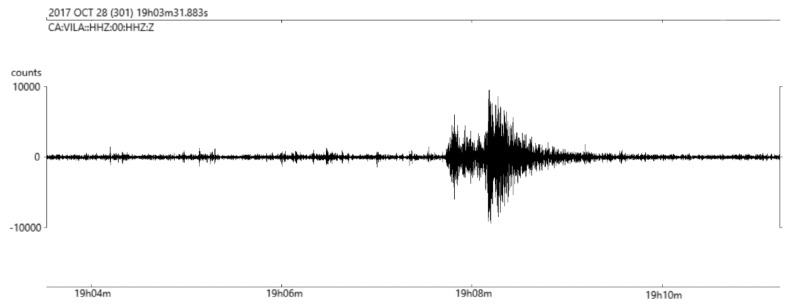
Caption from the Cabled Seismometer Station of OBSE of a seismic event located at Hautes-Pyrénées, of magnitude 3.7, that occurred on October 28 at 19.06 UTC [16]. A band filter between 4 and 10 Hz has been used to filter the raw seismic data.

**Figure 14:**
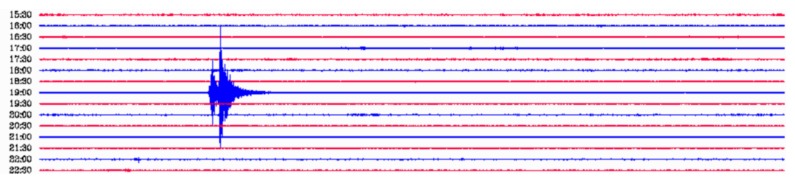
Caption from the Garraf Seismic Station of a seismic event located at Hautes-Pyrénées, of magnitude 3.7 [17], that occurred on 28 October at 19.06 UTC.

In order to validate this seismic event, we compared it with the seismic data from the Garraf Seismic Station [15] (see Figure 14), which is located at approximately 18.4 km from the OBSEA. Moreover, the moored OBS has been validated with the cabled seismometer station of OBSEA [16] which detected the same seismic event as illustrated in Figure 13. This cabled seismometer station is based on the Trillium 120P/PA broadband seismometer together with the Taurus data logger provided by Nanometrics Inc, and a GPS emulator developed with a Stellaris microcontroller [4].

## 4. Conclusions

In this paper we described a new technology of a moored OBS, which can help to meet the long-term goal of telemetered ocean bottom seismic stations. A seismometer attached to a surface buoy has been proposed as an alternative to classical OBSs. In contrast to autonomous OBSs, the physical connection between the seismometer and the buoy allows real-time data communications with enough bandwidth to analyse seismic activity. It should be noted that inductive systems, such as the one included in this mooring OBS, are relatively easy to implement with a single anchor point. Moreover, this telemetry system has a very low latency and power consumption compared to acoustic telemetry, which is normally used as communication link between the seafloor and surface devices. Having a moored OBS, such as the one described in this paper, installed offshore in an active seismic zone will allow us to obtain almost in real time the first record of an earthquake, and to determine the arrival time of the P wave and its amplitude. Hence, this type of system is able to provide crucial information over the ocean that global seismic tomography so far desperately lacks.

Two different mooring models, using a steel cable or a combination of steel cable plus elastomer, have been investigated. Numerical simulations, using Orcaflex, have been performed including the effect of the waves, winds, and currents, taken from the Spanish Harbor Authority records. Model 1 uses the inverse catenary to absorb movements and tensions whereas Model 2 uses an elastomer to do the same. The knowledge of these tensions is a key point in the design of the anchor system. Both models have pros and cons. Under normal conditions there is not a significant difference between models, although in extreme conditions Model 2 gives better results than Model 1, the seabed tension being about 4 times smaller. Under the studied conditions, the use of the elastomer reduced the tensions at the anchorage, minimizing the risk of displacement, and it seems to be a good option in extreme conditions.

Final design consists of a seafloor unit (Güralp 6YC-OBS Gimbal seismometer) and a surface unit (EMS 2.0 buoy) connected by a steel cable. The steel cable allows inductive DPSK data transmission between the two units and consequently real-time data transmission through RF, GSM, or satellite link to the land station. First deployment and validation tests were done in June of 2017 at 2 nautical miles off the coast of Vilanova i la Geltrú (Barcelona), near the OBSEA underwater observatory at a depth of 20 m. The reliability of the system has been proved by detecting a seismic event of magnitude 3.7 centered at Hautes-Pyrénées, that occurred on 28 October at 19.06 UTC. Finally, we plan to deploy the moored OBS system in an active seismic zone in the Carboneras Fault in the Alboran Sea (Western Mediterranean) at a depth of 250–300 m.

Usually seismic Long-Period events (LP) range from 0.05 to 30 Hz. Our design, in the studied meteorological conditions, transmits seabed vibrations around 0.1, 0.2, and 0.6 Hz that partially overlap the seismic range. Special attention must be taken in order to identify low frequency seismic events. The vibrations of the nearby anchor due to the wave can be induced in the signal of the geophone, however, this behaviour can be characterized using the new elastic approach with a simple mass-spring-dashpot model of OBS/seabed interaction, that allows the analysis of amplitude coupling data [18]. It is also possible to make corrections for this problem by the infidelity vector of the coupling [19]. Our dynamical study of the system and the possibility of segregating the seismic data using spectral analysis paves the way to a new generation of seismic buoys, more compact, lighter, and easier to deploy.

## Figures and Tables

**Table 1:** Lines configuration for the different models.

		Model 1	Model 2
**Line 1**	type	steel cable	elastomer
	length (m)	120/140/160	17/24/28
**Line 2**	type	steel cable	steel cable
	length (m)	200	280

**Table 2:** Meteorological conditions tested. A JONSWAP wave type is used. Hs (m) is the significant wave height; T_z_ (s) is the wave period.

Property		METEO		Units
		Normal	Extrem	
Wave data	Hs	1	5.7	m
	T_z_	3.73	8.61	s
Wind velocity		3	20	m/s
Current velocity	on surface	0.20	1.20	m/s
	at 100 m depth	0.053	0.31	m/s
	at 200 m depth	0.028	0.17	m/s
	on the seabed	0.022	0.14	m/s

**Table 3:** Some relevant outputs from OrcaFlex simulations. Minimum, maximum, and variation range (Δx) of the horizontal relative position x (m) of buoys from simulations of Orcaflex model.

Meteo	Line Configuration			Surface Buoy			Line 2	Seabed
	Ratio Line 1	Line 1	Line 2	min *x*	max *x*	Δx	Angle	max T
		m	m	m	m	m	degrees	kN
	Model 1	Steel Cable	Steel cable					
normal	1.2	120	200	66.9	79.1	12.2	3.55	2.10
	1.4	140	200	85.9	97.1	11.2	2.52	1.42
	1.6	160	200	101	113	12.1	2.36	1.37
extreme	1.2	120	200	113	132	19.2	22.6	25.9
	1.4	140	200	155	174	18.7	27.8	21.9
	1.6	160	200	188	207	19.0	31.3	19.6
	**Model 2**	**Elastomer**	**Steel Cable**					
normal	0.85	17	280	11.2	18.8	7.6	2.85	2.00
	1.2	24	280	24.8	33.0	8.2	3.50	2.20
	1.4	28	280	28.8	37.6	8.8	3.42	1.46
extreme	0.85	17	280	85.1	127	41.4	22.1	7.50
	1.2	24	280	108	145	36.9	24.4	5.80
	1.4	28	280	109	145	36.6	24.4	5.84
